# Utilisation of village health workers’ services for tuberculosis screening in Lesotho

**DOI:** 10.4102/safp.v64i1.5581

**Published:** 2022-09-21

**Authors:** Regina M. Thetsane, Motšelisi Mokhethi, Maseabata Ramathebane, Nthatisi Leseba

**Affiliations:** 1 Department of Business Administration, Faculty of Social Sciences, National University of Lesotho, Maseru, Lesotho; 2 Department of Pharmacy, Faculty of Health Sciences, National University of Lesotho, Maseru, Lesotho; 3 Department of Statistics and Demography, Faculty of Social Sciences, National University of Lesotho, Maseru, Lesotho

**Keywords:** village health workers, tuberculosis, TB screening, TB knowledge, VHWs’ services

## Abstract

**Background:**

Village health workers (VHWs) play an essential role because they extend the capacity of primary healthcare, particularly for developing countries. In Lesotho, VHWs are part of the primary healthcare connecting the community with clinics in their respective villages. They contribute to the prevention of the spread of tuberculosis (TB) within their catchment areas by encouraging communities to partake in TB screening. This study aimed at identifying factors associated with the utilisation of VHWs’ service to undertake TB screenings in Lesotho.

**Methods:**

This study emanates from the main study that used a cross-sectional descriptive design. A total of 19 health service areas (HSAs) comprised 17 catchment areas and two clinics, each randomly selected from the District Health Management Team (DHMT) and the Lesotho Flying Doctors Service (LFDS), respectively. A total of 2928 individual household members aged 15 and above were included in the study. Data analysis included descriptive and inferential statistics.

**Results:**

There were more female than male respondents, with a majority (77%) below 65 years of age. Tuberculosis knowledge of respondents was mostly on the TB symptoms and curability of TB, but they were less knowledgeable about the causes of TB. The use of VHWs’ services for TB screening was very low (23.3%).

**Conclusion:**

The study revealed that while respondents were to some extent knowledgeable about TB, their utilisation of VHWs’ services for TB screening varied with education level, having worked in South Africa and the household size at α = 0.01.

## Introduction

The use of village health workers’ (VHWs) service is an initiative meant to address the inadequate healthcare system by strengthening and expanding the global health workforce, particularly in developing countries.^1,2^ They are recognised as an effective and efficient intervention in the promotion of good health practices in the community.^3,4^ It is also acknowledged that VHWs play a significant part in improving health outcomes.^5,6,7^ In Lesotho, VHWs are part of the primary healthcare team under the supervision and guidance of clinic professionals in charge of the health centres.^8,9^ They connect the community with clinics in their respective villages.^5^ Their roles in their respective communities include encouraging communities to uptake tuberculosis (TB) screening, facilitating educational activities on HIV and providing the necessary support during treatment,^10,11^ exposing communities to nutrition and immunisation programmes.^10^

While evaluations of the VHW programme have been made,^12,13^ no evaluation of the utilisation of VHWs’ services in the fight against TB could be found. Lesotho has a high TB incidence rate, estimated at 611 cases per 10 000 population in 2018.^14,15^ In Lesotho, TB is not only among the top 10 causes of death but also second after HIV and AIDS.^15^ The high HIV prevalence could be fuelling the high TB incidence rate, because people living with HIV are more susceptible to TB.^16,17,18,19^ As TB screening is vital in the fight against the spread of the disease, it is important to evaluate the role of VHWs in encouraging community members to go for TB screening.

It is acknowledged that for VHWs to deliver impressive health services, they require sufficient information and understanding of how to assist in the provision of quality health treatment outputs.^3,5^ However, it is equally important to establish the success of VHWs in encouraging members of the community to utilise the services they offer with the aim to minimise the spread of TB by screening. Quantifying the efforts of VHWs by estimating the proportion of members of the community advised by the VHWs to uptake TB screening would help to improve the current strategies of VHWs in the fight against the spread of TB in their communities.

It has been established that when communities have comprehensive knowledge about HIV, they will cooperate with the efforts made in the fight against the HIV and AIDS pandemic, and they will better look after themselves.^1^ The same can be said about comprehensive TB knowledge. Improved TB knowledge should translate into improved health treatment outcomes. As TB screening is critical in preventing the spread of the disease, this study explains why it is important to establish to what extent VHWs are successful in encouraging community members within their catchment areas to go for TB screening.

It was established that 74.5% of community members in Lesotho were aware of their TB status.^7^ Of the members aware of their TB status, less than half (43.8%) reported ever participating in TB screening. Only one in five respondents who were screened for TB did so on the advice of the VHW, showing underutilisation of these important team players in primary healthcare. Furthermore, 56.2% of respondents who knew about TB had never been screened for TB. This is a large percentage to be ignored, and undoubtedly there is more work for the VHWs to help raise TB awareness and TB screening uptake. Failure to undertake TB screening means that TB transmission is highly likely among community members who come in contact with affected persons, even before the disease is fully blown, and it would have affected many other people. It is within this context that the present study is being undertaken. Therefore, this study is aimed at establishing factors associated with the utilisation of VHWs’ services for TB screening in Lesotho.

## Methods

### Design

The main study whose data were used for the present study was a quantitative descriptive study with cross-sectional design, using a semistructured questionnaire. Members of the community whose responses were used for the present study were from selected villages within the catchment of the selected clinics. Detailed methodology of selecting clinics and villages that participated in the study is provided in the baseline report of the main study.^7^

### Population, sampling and sample size

The study setting is the health service area (HSA) and district health management team (DHMT). Health service area is an old system of health-implementing bodies based on 17 hospitals; therefore, there are 17 HSAs, as there were 17 hospitals in the country. District health management team is a new health system implementing body which has replaced HSAs, and there are 10 DHMTs in the country. A sample was made up of 19 clinics, comprising 17 clinics drawn from 17 HSAs and two clinics, each randomly drawn from the DHMT and Lesotho Flying Doctors Service (LFDS), respectively. A total of 100 households were sampled per village; however, all households in the visited village were included, even if that meant exceeding the target for the study. If households were less than 100 in a village, the nearby village was also included in the study. A total of 8295 respondents were interviewed for the main study. A total of 2928 respondents who were aware of TB and had been screened made up the sample size of the present study.

### Outcome variable

The outcome variable was utilisation of VHWs’ services and was measured by asking community members to report on who advised them to test for TB, among other pieces of information collected. All household members aged 15 years and above who were aware of their TB status and had ever taken a TB screening test provided information on who had advised them. Village health workers’ advice for TB screening was the response variable of the study, coded 1 if TB screening was advised by the VHW and 0 if advised by self, community member, relative, nurse or doctor or screening was a requirement at work.

### Explanatory variables

Explanatory variables of this study are age, sex of respondent and that of household head, household size, marital status, education, experience of working in the Republic of South Africa (RSA) and family TB history. Diabetic and HIV status of respondents were also included in the analysis as known TB risk factors. Family TB history and TB knowledge regarding how it is spread (modes of spreading TB), the symptoms of a presenting patient (knowledge of TB symptoms) and its cause (TB cause) were the summary variables computed based on the collected data. Family TB history was coded 1 if at least one household member had been diagnosed with TB and 0 if no member had been diagnosed with TB. Knowledge of signs was coded 1 if at least five symptoms for a presenting TB patient were known and knowledge was considered adequate. If less than five symptoms were indicated, knowledge of signs was considered inadequate and coded 0. Modes of TB transmission was coded 1 if only the correct mode of TB transmission was known, 2 if both correct and incorrect modes were mentioned and 3 if only incorrect modes were mentioned. Cause of TB was coded 1 if the correct cause was mentioned, 2 if correct and incorrect causes were mentioned and 3 if only incorrect causes of TB were mentioned. The TB knowledge variable that was not a summary variable was curability of TB coded 0 if respondent was unaware that TB is curable and 1 if the curability of TB was known.

Tuberculosis knowledge variables were selected and included as study variables on the premise that comprehensive TB knowledge among community members would ease the work of the VHW,^1^ hence their likelihood of association with VHW advice for TB screening. Family TB history, household size (as proxy for household crowding), HIV and diabetes status were included in the study as known TB risk factors.^20^ Historically, Basotho men have worked in the mining industry of South Africa, although at reduced proportions since the end of apartheid. As mining is a TB risk factor,^21^ experience of working in South Africa was also included as a variable of interest in the study.

### Data collection

This study used a data set from a commissioned study by the Ministry of Health, Lesotho, conducted in 2019. Open Data Kit (ODK) was used to collect data. This is a data collection tool that has a server to accept all submitted data and make it available whenever needed. Open Data Kit facilitated the collection of data electronically. Once a questionnaire was completed, the data were sent to the server at the university. The data were exported into Excel for cleaning and the Statistical Package for the Social Sciences (SPSS) for analysis.

### Data analysis

Descriptive statistics were used to summarise the demographic information of respondents in the study. Information collected from household members aged 15 years and above formed the basis for the analysis. The percentage of respondents utilising VHW’s services for TB screening was calculated. The relationship between explanatory variables, namely age, sex of respondent and that of household head, household size, marital status, education, experience of working in RSA and family TB history and utilisation of VHWs’ services for TB screening were determined with Pearson chi-square statistics. A *p*-value of < 0.05 was considered to represent a significant association. Multivariate logistic regression was run to establish variables’ association with the utilisation of VHWs’ service for TB screening. If there was a significant correlation between explanatory variables, only one was entered into the model to avoid multicollinearity.

### Limitations of the study

No mycobacteriological tests were carried out to establish the TB status of respondents. Tuberculosis status was self-reported, based on the outcomes of TB screening tests. There was also no time frame of when TB screening was performed. Family TB history was reported by the household head with no time frame as to when the member was diagnosed with TB.

### Ethical considerations

Ethical approval was given by the Ethical Committee of the Lesotho Ministry of Health (reference ID73-2016). All respondents provided written informed consent to indicate that they consented to participate in the study. Respondents’ names were not used on any of the study-related documents.

## Results

### Baseline characteristics of respondents

There was a total of 2928 respondents. A third (33.9%) of respondents were male, while more than half (56.8%) resided in female-headed households, and 14% resided in a household with at least one member diagnosed with TB. The median age was 40, and 4 in 10 (38.9%) respondents were aged less than 40. Just above half (55%) of the respondents had completed primary education, and the majority (84.5%) had never worked in South Africa (Table 1).

**TABLE 1:** Baseline characteristics of respondents (*N* = 2928).

Variable	Category	Percentage	Frequency
Gender of respondent	Male	33.9	992
	Female	66.1	1936
Gender of household head	Male	43.2	1264
	Female	56.8	1664
Age group	15–39	38.6	1129
	40–64	38.6	1130
	≥ 65+	19.6	573
	Age not stated	3.3	96
Educational attainment	No education	9.7	283
	Primary	55.0	1609
	Secondary or higher	35.4	1036
Experience of working in RSA	Never worked	84.5	2474
	Worked in the past	12.6	370
	Currently working	2.9	84
Family TB history	None	86.3	2527
	At least one member diagnosed with TB	13.7	401

RSA, Republic of South Africa; TB, tuberculosis.

### Tuberculosis knowledge among respondents

Four variables were used to measure TB knowledge: curability of TB, what causes TB, knowledge of symptoms of a TB-presenting patient and modes of spreading TB. As shown in Table 2, 64.2% of respondents mentioned correct modes of spreading TB, while 1 in 5 (23.7%) were not sure by mentioning correct and wrong modes of spreading TB. Regarding curability of TB, 9 in 10 (90.3%) respondents knew that TB is curable, while 5.4% did not know that TB is curable. Knowledge of what causes TB and symptoms of TB were low. Only 1 in 10 (10.3%) respondents knew that TB is caused by bacteria, and 18.5% had adequate knowledge of the symptoms of TB.

**TABLE 2:** Tuberculosis knowledge among respondents (*N* = 2928).

Variable	Category	Percentage	Frequency
Curability of TB	Curable	90.3	2644
	Not curable	4.3	125
	Do not know	5.4	159
TB causes	Only correct cause mentioned	10.3	301
	Correct and wrong causes mentioned	34.2	1002
	Only wrong causes mentioned	31.3	915
	Do not know	24.2	710
Knowledge of TB symptoms	Inadequate knowledge	81.5	2387
	Adequate knowledge	18.5	541
Modes of spreading TB	Only correct mode mentioned	64.2	1879
	Correct and wrong modes mentioned	23.7	695
	Only wrong modes mentioned	4.2	122
	Do not know	7.9	232

TB, tuberculosis.

### Utilisation of village health worker’s services

Table 3 presents the percentage of respondents who utilised the services of the VHWs for TB screening. Overall, 23.3% of respondents used the services of VHWs to test for TB. However, there was a marginal difference between variables, except for a few where the reported percentage was at least 4% points below (˂ 19.3) or above (˃ 27.3). The highest utilisation of VHWs’ services for TB screening was among respondents who mentioned correct and wrong modes of spreading TB (28.3%), followed closely by respondents with adequate TB knowledge (28.1%). Respondents who mentioned only wrong modes of spreading TB (11.5%), those who were diabetic (12.2%), those who consider TB incurable (16.0%), those who worked in RSA (16.4%), those residing in households with at least one member diagnosed with TB (18.7%) and those with secondary education or higher (19.1%) used the VHWs’ services for TB screening the least.

**TABLE 3:** Percentage of respondents utilising the services of the village health workers for screening.

Variable	Category	Percentage	Pearson chi-square
Gender of household head	Male	25.4	5.30*
	Female	21.7	
Gender of respondent	Male	25.5	4.025*
	Female	22.2	
Marital status	Never married	22.5	0.179
	Currently married	23.4	
	Previously married	23.4	
Age group	< 40	23.5	5.409
	40–64	24.9	
	65+	19.9	
Educational attainment	No education	23.9	16.208**
	Primary	25.9	
	Secondary or better	19.1	
Experience of working in RSA	Never worked	24.4	13.648**
	Worked	16.4	
Diabetic status	No	23.5	3.402
	Yes	12.2	
Family TB history	None	24.0	5.469*
	At least one	18.7	
HIV status	Positive	21.2	3.695
	Negative	24.5	
Knowledge of curability of TB	Curable	23.8	4.059*
	Not curable	16.0	
Adequacy of knowledge of TB symptoms	Inadequate	22.2	8.594**
	Adequate	28.1	
Household size	< 5	26.8	22.901**
	≥ 5	22.5	
Knowledge of modes of spreading TB mentioned	Only correct mode	22.5	19.234*
	Correct and wrong modes	28.3	
	Only wrong modes	11.5	

**Overall**	**-**	**23.3**	**-**

RSA, Republic of South Africa; TB, tuberculosis; HIV, human immunodeficiency virus.

* , Significant at α = 0.05;

** , significant at α = 0.01.

Based on the Pearson chi-square statistic (Table 3), marital status, age, being diabetic and the HIV status variables did not suggest any association with utilisation of services for TB screening. However, nine variables, namely adequacy of knowledge of TB symptoms, education, experience of working in RSA, gender of respondent, gender of household head, household size, knowledge of curability of TB, knowledge of modes of spreading TB mentioned and family TB history suggested an association with utilisation of services for TB screening.

### Factors associated with the utilisation of services for tuberculosis screening

All variables that showed statistically significant association at the bivariate analysis level were entered into the conditional multivariate logistic regression analysis. As a result of significant correlation between TB knowledge variables, only adequacy of knowledge of TB symptoms was entered into the model, as it produced a better fit than the other two (knowledge of curability of TB and knowledge of modes of spreading TB mentioned). The correlation between the gender of the head of the household and the gender of the respondent was also statistically significant, but only the gender of the respondent was selected over the gender of the head for the same reasoning. As was the case at the bivariate level, all the variables were significantly associated with the use of VHWs’ services for TB screening.

Table 4 reflects that adequacy of knowledge of TB symptoms was significantly associated with the use of service for TB screening. It is more probable for respondents with adequate knowledge of TB symptoms to report the use of services for TB screening relative to their counterparts with inadequate or lack of knowledge (odds ratio [OR] = 1.398, confidence interval [CI]: 1.127, 1.733). Respondents residing in households with a history of TB were more likely to report the use of the services of the VHWs for TB screening relative to their counterparts residing in households without TB history (OR = 1.353, CI: 1.017, 1.800).

**TABLE 4:** Probability of using village health workers’ services for tuberculosis screening.

Variable	Category	B	*p*	OR	95% CI	
					Lower	Upper
Education	No education	0.139	0.413	1.149	0.824	1.603
	Primary	0.365	0.000	1.440	1.186	1.748
	Secondary or higher	-	-	-	-	-
RSA work experience	Never worked	0.563	0.000	1.756	1.332	2.316
	Worked	-	-	-	-	-
Family TB history	None	0.302	0.038	1.353	1.017	1.800
	At least one	-	-	-	-	-
Gender	Male	0.287	0.004	1.332	1.096	1.618
	Female	-	-	-	-	-
Adequacy knowledge of TB symptoms	Adequate	0.335	0.002	1.398	1.127	1.733
	Inadequate	-	-	-	-	-
Household size	< 5	0.310	0.001	1.363	1.136	1.635
	> 5	-	-	-	-	-
Constant	−2.532	0.000	0.079	-	-	-

OR, odds ratio; CI, confidence interval; TB, tuberculosis; RSA, Republic of South Africa.

With regard to education, it is more probable for respondents with primary school level or no education to report the use of services for TB screening (primary school level, OR = 1.440, CI: 1.186, 1.748; no education, OR = 1.149; CI: 0.824, 1.603), relative to their counterparts with secondary education and higher. Regarding gender, it is more probable for men to report using services for TB screening relative to their female counterparts (OR = 1.332; CI: 1.096, 1.618). It is also more probable for the respondents who had never worked in RSA to report using services for TB screening relative to their counterparts who had worked in RSA (OR = 1.756; CI: 1.332, 2.316). Households with fewer than five members were also more likely to report the use of the services for TB screening compared with their counterparts with five or more members (OR = 1.363; CI: 1.136, 1.635).

## Discussion

The results showed that TB knowledge variables were significantly associated with the use of the services for TB screening, supporting the importance of comprehensive TB knowledge. The results in this study support what has been found by other studies, suggesting that comprehensive TB knowledge is critical for TB screening.^22^

There is varying utilisation of VHWs’ services for TB screening for the demographic factors studied. In some cases, the results were consistent with previous studies, while in other incidences, the findings were contradictory.^23,24^ For instance, family TB history (those residing in households without TB history) results were contrary to expectation. Households with a history of family TB (those residing in households with TB history) should have had frequent visits from the VHW, particularly while on treatment to administer directly observed therapy (DOT). Usually, when a member of the household tests positive for TB, all other members are encouraged to go for TB screening by the VHW. Based on these facts, the use of the services of the VHWs for TB screening was expected to be high for households with a family TB history and not vice versa. The results do not reflect a good sign for VHWs, because it suggests that VHWs are not building a good rapport when working with the community members.

The utilisation of VHWs’ services by gender in this study do not support findings from other studies^22^ suggesting that the utilisation of health facility is commonly high among women compared with their male counterparts. In this study, men seemed to utilise the services more than women, but the difference reported was marginal. These results are consistent with the study that^25^ found that both men and women use the services of the VHWs at the same rate, in particular for TB screening. On the other hand, another study^22^ found that women generally use more VHWs’ services than men, particularly on issues related to pregnancy advice. As this study is focusing on the utilisation of the services of the VHWs on TB screening, it is not surprising that both gender groups seem to use the services at the same rate.

In addition, the results revealed varying utilisation of VHWs in relation to education levels, where respondents with primary education and below used VHWs’ service more than those with higher education levels. Also, people who have not worked in South Africa used VHWs’ service more than respondents who worked in South Africa. Lastly, the household size had a bearing on the utilisation of VHWs’ services, where families with fewer than five members used the VHWs’ services more than their counterparts with five or more members. Other studies also found varying results concerning the utilisation of VHWs’ services, where the well-informed community members self-referred themselves directly to other health centres in towns.^26^

### Implications and recommendation

In 2015, Lesotho did not meet the Millennium Development Goal for ending TB transmission and mortality. Lesotho remains among the 10 countries with the highest TB incidence rate.^14^ The country needs to use every opportunity to fight the spread of TB, and the VHW programme is one such strategy. Targeting certain groups within communities and encouraging them to go for TB screening through the VHW programme would go a long way in achieving Goal 3 of the Sustainable Development Goals. This study will be beneficial because it has revealed that the utilisation of VHWs’ services for TB screening is low (23.3%), and this is important information to policymakers as it will inform them to come up with strategies to improve the use of VHWs in TB screening. Also, the results of the study can be generalised to communities in Lesotho, as the sample was representative of the population.

## Conclusion

The study revealed that even if respondents are knowledgeable about TB, their response to the utilisation of VHWs’ services for TB screening will vary with some demographic factors. This study showed education level, work experience in South Africa and household size as factors associated with the use of VHWs’ services for TB screening for communities in Lesotho. On the other hand, gender of the respondents, gender of household head and family TB history did not show an association with the utilisation of VHWs’ services for TB screening.
