# Corrigendum: Utilisation of village health workers’ services for tuberculosis screening in Lesotho

**DOI:** 10.4102/safp.v65i1.5759

**Published:** 2023-07-20

**Authors:** Regina M. Thetsane, Motšelisi Mokhethi, Maseabata Ramathebane, Nthatisi Leseba

**Affiliations:** 1 Department of Business Administration, Faculty of Social Sciences, National University of Lesotho, Maseru, Lesotho; 2 Department of Pharmacy, Faculty of Health Sciences, National University of Lesotho, Maseru, Lesotho; 3 Department of Statistics and Demography, Faculty of Social Sciences, National University of Lesotho, Maseru, Lesotho

In the published article, Thetsane RM, Mokhethi M, Ramathebane M, Leseba N. Utilisation of village health workers’ services for tuberculosis screening in Lesotho. S Afr Fam Pract. 2022;64(1):a5581. https://doi.org/10.4102/safp.v64i1.5581, on page 4 and 5 the following paragraph is updated as it was incorrectly formulated:

**The original incorrect wording:**

Respondents residing in households with a history of TB were more likely to report the use of the services of the VHWs for TB screening relative to their counterparts residing in households without TB history (OR = 1.353, CI: 1.017, 1.800).

**The revised and updated wording:**

Respondents residing in households with no history of TB were more likely to report the use of the services of the VHWs for TB screening relative to their counterparts residing in households with TB history (OR = 1.353, CI: 1.017, 1.800).

Furthermore, on page 5 the following paragraph is updated as it was incorrectly formulated:

**The original incorrect wording:**

The study revealed that even if respondents are knowledgeable about TB, their response to the utilisation of VHWs’ services for TB screening will vary with some demographic factors. This study showed education level, work experience in South Africa and household size as factors associated with the use of VHWs’ services for TB screening for communities in Lesotho. On the other hand, gender of the respondents, gender of household head and family TB history did not show an association with the utilisation of VHWs’ services for TB screening.

**The revised and updated wording:**

The study revealed that the utilisation of VHWs’ services for TB screening will vary with some demographic factors. For instance, education level, work experience in South Africa and household size are some factors associated with the use of VHWs’ services for TB screening for communities in Lesotho. On the other hand, gender of the respondents, marital status and HIV status did not show an association with the utilisation of VHWs’ services for TB screening.

The authors apologise for this error. The correction does not change the study’s findings of significance or overall interpretation of the study’s results or the scientific conclusions of the article in any way.

